# No clinical advantage of locking over nonlocking plate fixation of symphyseal disruptions

**DOI:** 10.1590/0100-6991e-20213122

**Published:** 2021-12-10

**Authors:** CHRISTIANO SALIBA ULIANA, EIJI RAFAEL NAKAHASHI, LUIZ HENRIQUE PENTEADO SILVA, ANDERSON FREITAS, VINCENZO GIORDANO

**Affiliations:** 1 - Hospital do Trabalhador, Universidade Federal do Paraná, Ortopedia - Curitiba - PR - Brasil; 2 - Instituto de Ortopedia e Traumatologia, Ortopedia - Passo Fundo - RS - Brasil; 3 - Hospital de Ortopedia e Medicina Especializada (HOME), Instituto de Pesquisa e Ensino - Brasília - DF - Brasil; 4 - Hospital Regional do Gama, Ortopedia - Brasília - DF - Brasil; 5 - Hospital Municipal Miguel Couto, Serviço de Ortopedia e Traumatologia Prof. Nova Monteiro - Rio de Janeiro - RJ - Brasil; 6 - Clínica São Vicente, Rede D’or São Luiz, Ortopedia - Rio de Janeiro - RJ - Brasil

**Keywords:** Pubic Symphysis Diastasis, Fracture Fixation, Internal, Pain, Postoperative, Treatment Failure, Quality of Life, Sínfise Pubiana, Fixação Interna de Fraturas, Dor Pós-Operatória, Falha de Tratamento, Qualidade de Vida

## Abstract

**Purpose:**

although locking plates have led to important changes in fracture management, becoming important tools in the orthopedic surgeon’s arsenal, the benefits of locking plates for traumatic diastasis of the pubic symphysis have not been established. This study was conducted to assess the quality of life in its different domains among patients with traumatic diastasis of the pubic symphysis managed either with locking or nonlocking plate.

**Methods:**

a prospective cohort study was undertaken at 3 level 1 trauma centres in Brazil. Patients presenting traumatic diastasis of the pubic symphysis treated with plate fixation with a minimum follow-up of 12 months were eligible for inclusion. Through a Pfannenstiel approach, the pubic symphysis was reduced and fixed with a superiorly positioned 4.5mm four to six hole reconstruction locked plate or 3.5mm four to six hole reconstruction nonlocked plate. Posterior injury was managed during the same procedure. Outcome measures were adequate healing of the pelvic injuries, return to pre-injury level on daily activities, and quality of life at the last follow-up visit. Complications and modes of failure were summarized and reviewed. Bivariate linear regression was used to assess individual factors affecting patients’ health-related quality of life. A p value of <5% was considered significant.

**Results:**

a total of 31 adult patients (29 males and 2 females) were eligible for the study. Thirteen patients were managed with a reconstruction locked plate and 18 patients with a nonlocked reconstruction plate. Average postoperative follow-up time was 24 months. Adequate healing of the pelvic injuries was achieved in 61.5% of patients treated with locking plates and 94.4% of patients treated with nonlocking plates (p=0.003). Radiographic failure of fixation with minor complications occurred in 46.1% of patients after locked plating versus 11.1% of patients in the nonlocking plate group (p=0.0003). In bivariate analysis, abnormal gait (p=0.007) was associated with a reduced long-term quality of life as measured with the EQ-5D-3L.

**Conclusion:**

internal fixation of traumatic diastasis of the pubic symphysis with locking plates has no clinical advantage when compared to nonlocked plating. Mechanical failure and inadequate healing are significantly increased after locked plating of the pubic symphysis. Therefore, we do not recommend routine use of locking plates for managing patients presenting traumatic diastasis of the pubic symphysis.

**Level of evidence:**

II (prospective, cohort study).

## RESUMO

**Justificativa e Objetivo:**

embora as placas bloqueadas tenham levado a mudanças importantes no tratamento de fraturas, tornando-se ferramentas importantes no arsenal do cirurgião ortopédico, os benefícios para a fixação da lesão da sínfise púbica não foram adequadamente estabelecidos. Este estudo foi realizado para avaliar a qualidade de vida em diferentes domínios de pacientes com disjunção traumática da sínfise púbica tratados com placas bloqueadas e não bloqueadas.

**Métodos:**

trata-se de estudo de coorte prospectivo, realizado em três centros de trauma nível 1, no Brasil. Foram elegíveis para inclusão no estudo pacientes com disjunção traumática da sínfise púbica tratados com redução aberta e fixação interna com placa, com seguimento mínimo de 12 meses. Por meio de abordagem de Pfannenstiel, a sínfise púbica foi reduzida e fixada com uma placa bloqueada de reconstrução de 4,5mm de quatro a seis orifícios posicionada superiormente ou com uma placa não bloqueada de reconstrução de 3,5mm de quatro a seis orifícios. A lesão pélvica posterior foi tratada durante o mesmo procedimento. Os desfechos analisados na última visita de acompanhamento foram cicatrização da lesão pélvica, retorno às atividades diárias para nível pré-lesional e qualidade de vida. Complicações e modos de falha foram observados e descritos. Foi utilizada regressão linear bivariada na avaliação dos fatores individuais que afetaram a qualidade de vida relacionada à saúde dos pacientes, com valor p <5% considerado significativo.

**Resultados:**

foram incluídos no estudo 31 pacientes adultos (29 homens e 2 mulheres). Treze pacientes foram tratados com placa de reconstrução bloqueada e 18 com placa de reconstrução não bloqueada. O tempo médio de seguimento pós-operatório foi de 24 meses. A cicatrização adequada da lesão do anel pélvico foi alcançada em 61,5% dos pacientes tratados com placas bloqueadas e em 94,4% dos pacientes tratados com placas não bloqueadas (p=0,003). Falha radiográfica de fixação com complicações menores ocorreu em 46,1% dos pacientes tratados com placa bloqueada contra 11,1% dos pacientes no grupo de placas não bloqueadas (p=0,0003). Na análise bivariada, marcha anormal (p=0,007) foi associada à redução da qualidade de vida em longo prazo, medida com o EQ-5D-3L, embora não tenha sido observada relação direta destas com os implantes utilizados.

**Conclusão:**

a fixação interna da disjunção traumática da sínfise púbica com placas bloqueadas não apresenta vantagem clínica quando comparada com placas não bloqueadas. Falha mecânica e cicatrização inadequada aumentam significativamente após o uso de placas bloqueadas na sínfise púbica. Portanto, não recomendamos o uso rotineiro de placas bloqueadas para o tratamento de pacientes com disjunção traumática da sínfise púbica.

**Nível de evidência:**

II (estudo de coorte prospectivo).

## INTRODUCTION

Locking plates have led to important changes in fracture management, becoming important tools in the orthopedic surgeon’s arsenal^1^ ^,^ ^2^. The biomechanical principles and design characteristics of the locking plate fixation promote biological stabilization while improving fixation by converting the shear stress created during loading into compressive stress at the screw interface^1^ ^,^ ^3^. Moreover, the introduction of locking plates coincided with the development of minimally invasive fracture fixation approaches^1^ ^,^ ^4^. Recognizing the general indications for use of locking plates and the potential complications resulting of implant misapplications are critical for a successful outcome^2^ ^,^ ^5^ ^,^ ^6^. Insufficient preoperative planning, such as the definition of the correct length and strength of the construct, has been pointed out as the main reason for failure, particularly when minimally invasive surgery is attempted, highlighting the importance of adhering to well-established principles of operative fracture management and learning how to maximize its clinical efficacy in each specific body region^2^ ^,^ ^7^ ^,^ ^8^.

Nowadays, the benefits of locking plates for traumatic diastasis of the pubic symphysis have not been established^9^. A recent retrospective analysis of a prospectively database from a single center showed that the use of locking plates for pubic symphysis diastasis is safe and effective in allowing patients to weight bear early, with a low complication rate and need for re-operation^10^. However, many authors have demonstrated no biomechanical advantage of locking plates in stabilizing the pubic symphysis over nonlocking implants in unstable pelvic ring injuries^9^ ^,^ ^11^ ^-^ ^13^.

So far, specific indications for the use of locking plates for the pubic symphysis remain to be determined. To the best of our knowledge, there is no prospective clinical study comparing the outcome of patients treated with locking versus nonlocking plate fixation for traumatic diastasis of the pubic symphysis. We hypothesized that there would be no clinical benefit of internal fixation of a traumatic diastasis of the pubic symphysis with locking plates compared to nonlocking implants. This study was conducted to assess the quality of life in its different domains among patients with traumatic diastasis of the pubic symphysis managed either with locking or nonlocking plate.

## METHODS

### Patients and preoperative screening

This is a prospective cohort study carried out in three level I trauma centers in Brazil, one university hospital and two regional hospitals. Adult patients with traumatic pubic symphysis disjunction treated with open reduction and fixation with a reconstruction plate, with a minimum follow-up of 12 months, were included. Patients with incomplete medical records or those treated with different fixation methods for the anterior pelvic lesion, such as rami lag-screws or double plate fixation, were excluded from the study. The study was approved by the Research Ethics Committee under number 3.397.602, with a substantiated opinion from the institutions, and informed consent was obtained from all patients.

On admission to the hospital, patients were primarily evaluated and clinically and hemodynamically stabilized. Secondary evaluation included images of the pelvic injury with radiographs in the anteroposterior (AP), inlet and outlet views, and computed tomography. Pelvic injuries were classified using the Young and Burgess classification system^14^. Preoperative evaluation included patient-specific data, past medical history, physical examination, and laboratory and imaging studies. The mechanism of trauma and associated injuries were recorded.

### Surgical procedure and in-hospital management

Patients were operated on with intravenous (IV) antibiotic administration 30 minutes before surgery. A Foley catheter was used to empty the bladder before the operation, thus reducing the risk of iatrogenic bladder injury. Using a radiolucent table, a Pfannenstiel approach was routinely performed to reduce the symphysis pubis, avoiding anterior dissection of the pubis. All operations were performed by one of three surgeons (CSU, LHPS, VG). Symphysis fixation was performed using a 4.5mm locked reconstruction plate (Synthes, Paoli, USA) with four to six holes (Group 1) or a 3.5mm unlocked reconstruction plate (Ostosintese, Jaraguá, Brazil) with four to six holes (Group 2). The plate was always positioned superiorly to the pubic rami, allowing the placement of screws from cranial to caudal. The criteria for choosing the type of used plate depended on availability, with no randomization or choice by the surgeon. Posterior injury was managed during the same procedure.

In the postoperative period patients received broad-spectrum prophylactic IV antibiotic for 24 hours. Pharmacological thromboprophylaxis with 40mg subcutaneous enoxaparin was given for three weeks. Mechanical thromboprophylaxis was encouraged with active and passive joint mobilization, muscular wasting, and flat-foot weight bearing as tolerated using two crutches or a supportive walker.

### Outcome measures

After discharge, patients were seen as outpatient at three, six, and 12 weeks, six and 12 months, and once per semester after the first year. Outcome measures were (i) adequate healing of the pelvic injuries, (ii) return to pre-injury level on daily activities, and (iii) quality of life at the last follow-up visit. During the follow-up visits, clinical and radiographic evaluations were used to allow patients to progressively increase weight bearing and return to their full activities. Adequate healing of the pelvic injuries was defined as low pain level and static radiographic findings on sequential follow-up radiographs more than six weeks apart at six months post-injury^15^.

Complications and modes of failure were observed and documented. Complications were classified as major, defined as complete loss of the anterior pelvic fixation requiring reoperation, and minor, defined as loosening of screws, broken screws, or broken plates not requiring any further surgical procedure. Return to the pre-injury level to daily activities was assessed according to the using a modification of the proposed criteria by Peek et al. modified^16^, as “definitely unable to return”, “ able, but not to the pre-injury level” and “at the same level as before the injury”. Quality of life was assessed using the EuroQol 5-dimensional 3-level questionnaire (EQ-5D-3L)^17^ ^,^ ^18^, which consists of a descriptive system covering five dimensions (mobility, self-care, usual activities, pain/discomfort, and anxiety/depression) with three levels in each (no problems, moderate problems, and extreme problems).

### Statistical analysis

Data were presented using absolute numbers with percentages (%) for dichotomous and categorical variables. Bivariate linear regression was used to assess individual factors affecting patients’ health-related quality of life. A p value of <5% was considered significant.

## RESULTS

A total of 31 adult patients (29 men and 2 women) were eligible for the study. Mean age was 36.1 years (ranging from 16-64 years). Twenty-two injuries were classified as type II anteroposterior compression (APC), six injuries as type III APC, and three injuries as vertical shear (VS). Two (6.4%) patients had bilateral injuries. Age, sex, and type of injury did not differ significantly between groups (p>0.05). Patient demographic information and injury characteristics are presented in Table 1.

**Table 1:** Demographic information of patients and characteristics of the lesions.

Patient	Gender	Age (in years)	Mechanism of injury	Classification	Associated injuries
1	M	54	Hit by car	APC-II	Y
2	M	16	Fall from bicycle	APC-II	Y
3	M	34	MVA	APC-II	Y
4	M	22	MCA	APC-II	Y
5	M	34	MCA	APC-II	N
6	M	49	MCA	APC-II	Y
7	M	37	MCA	APC-II	N
8	M	57	MCA	APC-II	Y
9	M	37	MCA	APC-II	Y
10	M	47	Fall from horse	APC-II	N
11	M	28	Fall from motorcycle	APC-III	N
12	M	24	MCA	APC-II	N
13	M	62	Hit by motorcycle	APC-II	Y
14	M	60	Fall from scaffold	APC-III	N
Patient	Gender	Age (in years)	Mechanism of injury	Classification	Associated injuries
15	M	34	Fall from motorcycle	APC-II	N
16	M	40	Fall from motorcycle	APC-III	N
17	M	27	Crushed by car	VS	Y
18	F	22	Hit by car	APC-II	Y
19	M	27	MCA	APC-III	Y
20	M	16	Fall from motorcycle	APC-II	Y
21	M	36	MVA	VS	N
22	M	31	MCA	VS R / APC-III L	N
23	M	32	Fall from scaffold	APC-II	Y
24	M	28	Fall from motorcycle	APC-II	Y
25	M	32	Fall from motorcycle	APC-II	Y
26	M	27	Fall from motorcycle	APC-II bilateral	N
27	M	30	Fall from motorcycle	APC-III	N
28	M	40	Hit by car	APC-II	Y
29	M	64	Hit by car	APC-II	Y
30	F	33	Fall from motorcycle	APC-II	N
31	M	39	MCA	APC-III	Y

Source: HT, IOT, and HMMC, 2020. Legends: M - male; F - female; MVA - motor vehicle accident; MCA - motorcycle accident; APC - antero-posterior compression; VS - vertical shearing; R - right; L - left; Y - yes; N - no.

Thirteen patients had the symphysis disruption stabilized with a 4.5mm locked reconstruction plate (Group 1) and 18 patients with a 3.5mm unlocked reconstruction plate (Group 2). Posterior fixation was performed with one or two sacroiliac or transiliac screws percutaneously (n=27), double anterior plate using an Olerud approach (n=3), or a transiliac posterior tension-band plate (n=1).

**Figure 1:**
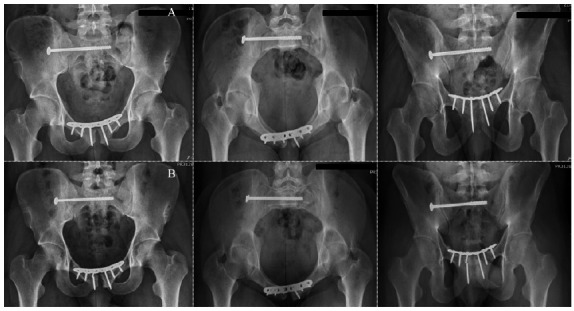
A. Postoperative radiographic views in AP, inlet, and outlet of the pelvis of a 22-year-old male patient who suffered type II APC injury after a motorcycle accident. The patient was operated on, and the pubic symphysis was fixed with a 4.5mm locked reconstruction plate. The sacroiliac joint injury was treated with a 7.0mm cannulated screw and washer in S1. B. AP, inlet, and outlet radiographs of the same patient taken at the last outpatient follow-up assessment showing adequate healing of the pelvic lesion despite a broken screw on the right side of the pubic symphysis.

The healing of the pubic symphysis injury was considered adequate in eight (61.5%) patients in Group 1 and in 17 (94.4%) patients in Group 2 (p=0.003). There were no cases of surgical wound infection. Radiographic fixation failure with minor complications occurred in six (46.1%) patients in Group 1, four (30.8%) due to screw loosening and two (15.4%) due to screw breakage, and in two (11.1%) patients in Group 2 (p=0.0003), both due to loosening of the implant (Figure 1). There were no major complications in both groups. No patients required implant removal or revision operation.

Mean postoperative follow-up time was 24 months (ranging from 12-40 months), with no significant difference between groups (p>0.05). According to Peek et al. modified criteria, nine (69.2%) patients in Group 1 and 14 (77.8%) in Group 2 reported being “able to perform daily tasks, but not at the same pre-injury level” or “able to perform daily tasks at the same level as before the injury” (p>0.05). Four (30.8%) patients in Group 1 and 4 (22.2%) in Group 2 reported moderate problems related to mobility and usual activities (p=0.078). All patients with gait disorders required some type of support device for walking, such as canes, crutches or walkers, or even the use of ankle-foot orthosis. No patients reported “no problems” or “extreme problems” in all five dimensions of the EQ-5D-3L. In the bivariate analysis, gait abnormality (p=0.007) was directly associated with reduced long-term quality of life as measured by the EQ-5D-3L.

## DISCUSSION

In the present study, there was no advantage of the internal fixation of the traumatic diastasis of the pubic symphysis with locked plates compared to non-locked implants. There was a significant increase in the risk of mechanical failure and clinical complaints after osteosynthesis of the pubic symphysis with a locked plate. As a result, adequate healing of the anterior pelvic injury was seen in only 61.5% of patients treated with locked plates versus 94.4% of those treated with unlocked plates.

Our findings are consistent with what has been reported clinically and biomechanically about the use of the locked plate for fixation of the pubic symphysis disruption. Although, in general, locked plate systems have greater mechanical strength to anteroposterior shear than non-locked plates, there is a higher rate of early loosening and risk of osteosynthesis failure^9^ ^,^ ^11^ ^-^ ^13^ ^,^ ^19^ ^,^ ^20^. This is probably because the physiological movements and deforming forces in the pubic symphysis are still not clearly understood, making the amount of rigidity of the pubic symphysis-plate construct an open question^15^ ^,^ ^21^ ^,^ ^22^. In the present study, 46.1% of the patients had pubic symphysis fixation failure after osteosynthesis with a locked plate, against 11.1% of failures in patients fixed with an unlocked plate. Our finding can be explained, at least partially, by the greater capacity to support cyclic loads when the pubic symphysis is fixed more flexibly compared to more rigid constructions^23^. Therefore, it seems reasonable to choose an unlocked implant for fixation of the traumatic injury to the pubic symphysis. Interestingly, it has been shown that more than 30% of patients undergoing osteosynthesis of the pubic symphysis with a plate, regardless of the type of implant used, show radiological signs of loosening of the implant during the first postoperative year, with loose or broken screws^15^ ^,^ ^24^. However, surgical revision is rarely indicated for removal of implants or revision of osteosynthesis, and most patients return to their previous level of daily activities^15^ ^,^ ^24^ ^-^ ^26^.

The length of the unlocked plate remains a matter of debate. Previous studies have suggested that fixation of the pubic symphysis with an unlocked two-hole plate satisfactorily restores the anterior component of the pelvic ring, allowing for physiological movements of the pubic symphysis^27^ ^,^ ^28^. However, more recent Sagi and Papp showed a significantly higher rate of fixation failure and malunion after pubic symphysis osteosynthesis with a two-hole unlocked plate compared to longer unlocked plates, suggesting that excessive movement before ligament healing produces supraphysiological load, leading to hemipelvis displacement^25^. Other authors have shown that, in addition to the use of longer unlocked plates, adequate restoration of pelvic ring congruence and stabilization of the sacroiliac joint, even in rotationally unstable lesions, decrease the rate of fixation failure and healing problems of the anterior component^20^ ^,^ ^24^ ^,^ ^29^ ^,^ ^30^. This finding confirms the evidence that a degree of physiological anterior pelvic movement may be desirable, provided there is sufficient posterior support^20^ ^,^ ^29^. In our study, regardless of the type of pubic symphysis fixation, the posterior injury was fixed in all patients, which may be implied in the absence of major complications, including implant removal and revision operation.

In the present study, gait abnormality was associated with reduced long-term quality of life as measured by the EQ-5D-3L. According to modified criteria by Peek et al., 69.2% of patients in Group 1 and 77.8% in Group 2 saying they were at the same level or almost at the same level as before the injury, and no patients in both groups reported extreme problems in all five dimensions of the EQ-5D-3L. Some authors have reported that, despite an anatomical reconstruction of the pelvic ring, many patients are unable to recover their pre-injury functional and quality of life level^18^ ^,^ ^21^ ^,^ ^31^ ^,^ ^32^. Borg et al. found substantially lower quality of life in the physical and mental domains after two years of follow-up of patients undergoing pelvic ring fixation, who had radiological results considered satisfactory^31^. Several factors have been related to unfavorable outcomes that lead to decreased quality of life in these patients despite adequate reduction and stabilization of the pelvis, including sexual dysfunction, dyspareunia, persistent urinary and fecal incontinence, chronic low back pain, cosmetic dissatisfaction, and post-traumatic stress syndrome^18^ ^,^ ^31^ ^-^ ^36^. In the current study we did not observe a direct relationship between the used implants in the fixation of the pubic symphysis and the occurrence of any of these factors. 

The main strength of our study is its prospective character, which allowed to evaluate in a controlled way two groups that are very similar demographically, and to adopt a standardized surgical technique, basically differing in the fixation implant used in pubic symphysis injuries, whether locked or non-locked. On the other hand, some limitations can be observed in the current design, in particular the use of a non-specific locking plate system for the fixation of the anterior pelvic lesion, the lack of randomization for the inclusion of patients in both groups, with this definition being dependent the availability of the locked implant in only one of the hospitals participating in the study, and the small number of patients, which potentially reduces the impact of our findings. Nevertheless, our findings did not show any clinical advantage of internal fixation of the traumatic disruption of the pubic symphysis with locked plates compared to unlocked implants, which confirms previous observations by other authors.

## CONCLUSION

Internal fixation of the traumatic symphysis pubis disruption with locked plates has no clinical advantage when compared with unlocked plates. Mechanical failure of fixation and inadequate healing are significantly increased after the use of locked implants in the pubic symphysis. Therefore, we do not recommend the routine use of locked plates for the treatment of patients with traumatic pubic symphysis disjunction.
